# Parkinson's disease or atypical parkinsonism? The importance of acoustic voice analysis in differential diagnosis of speech disorders

**DOI:** 10.1002/brb3.1700

**Published:** 2020-06-11

**Authors:** Renata Kowalska‐Taczanowska, Andrzej Friedman, Dariusz Koziorowski

**Affiliations:** ^1^ Department of Neurology, The Faculty of Health Sciences Medical University of Warsaw Warsaw Poland

**Keywords:** acoustic voice analysis, atypical parkinsonism, dysarthria, Parkinson's disease

## Abstract

**Introduction:**

Speech disorder is a common clinical manifestation in patients with Parkinson's disease and atypical parkinsonian syndromes and tends to occur before the onset of the axial parkinsonian symptoms. Due to parkinsonian features that overlap those of Parkinson's disease, the differentiation of voice and a speech disorder is a challenge for clinicians primarily in the early stage of the disease.

**Methods:**

Speech samples were obtained from 116 subjects including 30 cases of Parkinson's disease, 30 cases of progressive supranuclear palsy, 30 cases of multiple system atrophy, and control group consisted of 26 subjects. Differential diagnosis of dysarthria subtypes was based on the quantitative, acoustic analysis of particular speech components. Additionally, Voice Handicap Index questionnaire was taken into account to differentiate the severity of voice impairment in the study groups.

**Results:**

Our results showed significant differences in the distribution of acoustic parameters between Parkinson's disease and atypical parkinsonian syndromes. A mixed type of dysarthria with a combination of hypokinetic, spastic, and atactic features has been found in patients with atypical parkinsonism. In patients with the clinical diagnosis of the parkinsonian variant of multiple system atrophy, ataxic components of dysarthria were observed. Patients with PD presented pure hypokinetic dysarthria. Some parameters may be used as a marker for the diagnosis of the initial stage of PD. Voice impartment was significantly more frequent and severe in atypical parkinsonism than in Parkinson's disease.

**Conclusion:**

Acoustic voice analysis is a very sensitive and noninvasive tool, provides objective information for the assessment of different speech components, has the specific potential to provide quantitative data essential for the improvement of the diagnostic process, and maybe a useful instrument in the differential diagnosis of parkinsonian syndromes.

## Graphical abstract

The aim of the study was to assess objectively by parametric measurements, speech, and voice disorders in hypokinetic, ataxic, and spastic dysarthria in PD and APS patients. Acoustic analysis can be diagnostically helpful in differential diagnosis of speech disorders in parkinsonian syndromes and may provide objective measures of treatment response and disease progression.

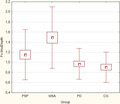

## 1 INTRODUCTION

Differentiation between Parkinson's disease (PD) and the atypical parkinsonian syndromes, such as progressive supranuclear palsy (PSP) and multiple system atrophy (MSA), is difficult, especially in initial stages when the clinical picture may be similar.

Parkinson's disease is a chronic and progressive neurodegenerative multisystem disorder, which causes damage to the dopaminergic neurons in the basal ganglia. Basal ganglia dysfunction implies the appearance of clinical symptoms in the form of bradykinesia, muscle rigidity, resting tremor, and postural instability (Berardelli et al., 2013; Darley, Aronson, & Brown, 1969a). Motor symptoms can often be preceded by the presence of nonmotor signs diagnosed as mood, behavioral, and cognitive disorder even a few years before the onset of the disease (Poewe, 2008). Most of the PD patients develop speech abnormalities defined as hypokinetic dysarthria described by mono‐pitch and mono‐loudness, variable rate, imprecise articulation, speech dysfluencies, inappropriate silence, reduced stress, and harsh voice quality (Darley et al., 1969a; Darley, Aronson, & Brown, 1969b; Logemann, Fisher, Boshes, & Blonsky, 1978). The previous study showed some form of vocal impairment in the early stages of the disease (Ho, Iansek, Marigliani, Bradshaw, & Gates, 1999; Logemann et al., 1978; Rusz, Cmejla, Ruzickova, & Ruzicka, 2011), and prosody turned out to be the most often affected speech subsystem in early untreated PD patients (Rusz et al., 2011).

Atypical Parkinsonian/Parkinson‐plus syndromes (APS), such as MSA and PSP, are relatively uncommon. The prevalence rate of PSP is 1.4–6.4/100,000 of the population and that of MSA 1.9–4.9/100,000 population (Schrag, Ben‐Shlomo, & Quinn, 1999; Tison, Yekhlef, Chrysostome, & Sourgen, 2000). Patients with APS develop parkinsonian features that overlap those of PD (Huh et al., 2015; Rusz et al., 2015; Sachin et al., 2008), especially in the disease onset thus many of them are initially diagnosed as suffering from PD (Hlavnička et al., 2017; Huh et al., 2015; Osaki et al., 2004; Rusz et al., 2015). Extensive involvement of the central nervous system structures (the basal nuclei, i.e, the globus pallidus, striate nucleus, subthalamic nucleus, pons, superior and middle cerebellar peduncles, dentate nucleus, cerebellum) yields additional clinical signs, resulting in the escalation of symptoms, more rapid disease progression and poor levodopa response (Nath, Ben‐Shlomo, Thomson, Lees, & Burn, 2003; O'Sullivan et al., 2008; Rusz et al., 2015). The cortico‐ponto‐cerebellar tract plays an important role in the development of speech disorders in APS (O'Sullivan et al., 2008; Tykalova, Rusz, Klempir, Cmejla, & Ruzicka, 2017). The differential diagnosis of PD and APS is based on patient history and clinical assessment guided by the diagnostic criteria established for specific syndromes, but it is not possible to completely eliminate all diagnostic errors (Berardelli et al., 2013; Gilman et al., 2008; Höglinger et al., 2017).

The involvement of the basal ganglia, corticobulbar pathways, and the cerebellum causes various symptoms of speech disorders. Routine assessment relies essentially on the qualitative description of the symptoms, and the differential diagnosis of speech impairment in parkinsonian syndromes especially during early stages of the disease may pose many difficulties. The hearing‐perceptual assessment allows the examiner to distinguish the characteristic PSP features, most importantly articulatory phenomena and speech functions. Palilalia (involuntary repetition of own syllables, words, or even whole phrases), echolalia (automatic repetition of vocalizations made by another person) and to a lesser extent stuttering are often observed although they are not pathognomonic. Moreover, in PSP patients, verbal expression is generally limited and nonspontaneous. Patients usually have word‐finding difficulties and at times nonfluent variant primary progressive aphasia (nfvPPA) may be observed (Kim & McCann, 2015). APS patients usually evolve mixed dysarthria with varied combinations of hypokinetic, spastic, and ataxic components with predominant of hypokinetic‐spastic features in the case of PSP, and hypokinetic‐ataxic signs in the case of MSA (Darley et al., 1969a; Hartelius, Gustavsson, Astrand, & Holmberg, 2006; Kluin, Foster, Berent, & Gilman, 1993; Kluin et al., 2001; Kluin, Gilman, Lohman, & Junck, 1996; Rusz et al., 2015; Rusz, Tykalowa, & Salerno, 2019; Sachin et al., 2008; Skodda, Visser, & Schlegel, 2011). Speech in PSP is characterized as strained‐strangled voice quality, stuttering like behavior, palilalia, variable rate of speech, and mono‐loudness (Kluin et al., 1993). Patients with MSA usually develop speech abnormalities described as mono‐pitch, excess pitch fluctuations, variable rate and loudness, imprecise consonants, and strained‐strangled voice quality (Hartelius et al., 2006; Kluin et al., 1996).

As confirmed by database search, the subjective assessment of speech disorders in PD, PSP, and MSA is well documented (Darley et al., 1969a, 1969b; Hartelius et al., 2006; Kluin et al., 1993, 1996; Logemann et al., 1978) whereas studies reporting data obtained using quantifiable measurements and describing differentiating PD from atypical parkinsonian syndromes based on speech assessment are scarce (Huh et al., 2015; Rusz et al., 2015, 2019; Skodda, Gronheitet, & Schlegel, 2012; Warnecke et al., 2019). The aim of the study was the quantitative assessment of speech dimensions and differential diagnosis of dysarthria based on the voice acoustic analysis.

## 2 MATERIALS AND METHODS

### 2.1 Participants

A total of 116 subjects were included in the study: 30 subjects with idiopathic Parkinson's disease (13 females, 17 males), 30 with the clinical diagnosis of PSP (9 females, 21 males), and 30 with the clinical diagnosis of parkinsonian variant MSA (MSA‐P) (19 females, 11 males) (Table 1). In the PSP group, 25 subjects were diagnosed with the Richardson's syndrome (PSP‐RS), 4 with predominant parkinsonism (PSP‐P), and one with corticobasal syndrome (PSP‐CBS). The diagnoses were based on patient history and clinical assessment fulfilling the established diagnostic criteria: the United Kingdom Parkinson's Disease Society Brain Bank for PD, the National Institute of Neurological Disorders and Stroke Society for PSP (NINDS‐SPSP) for PSP, and the “Second consensus statement on the diagnosis of multiple system atrophy” for MSA (Berardelli et al., 2013; Gilman et al., 2008; Höglinger et al., 2017). To exclude other diseases, all subjects underwent a neuroimaging study (magnetic resonance imaging, MRI). The disease duration was estimated based on the self‐reported occurrence of the first symptoms. MSA and PSP patients received levodopa before hospitalization with no benefit or poor response. None of the patients had psychotic episodes and received antipsychotic medication. To exclude possible pharmacological influence, all PD, MSA, and PSP patients were examined in off‐state, at least 12 hr overnight withdrawal of dopaminergic medication. In PD, the only patients staging below 3 on the Hoehn & Yahr scale were enrolled. The control group (CG) consisted of 26 subjects (13 female, mean age 63.1 and 13 male, mean age 64.6), without any neurological or laryngological disorders, who had had no surgery or other medical procedures involving the head and neck.

**TABLE 1:** Demographic data

2015–2019	PD *n* = 30 Mean/*SD*/range	PSP *n* = 30 Mean/*SD*/range	MSA *n* = 30 Mean/*SD*/range	*p* value
Sex F:M	13:17	9:21	19:11	n.s.
Age (years)	53.6/12.2//26.8–73.3	67.2/6.6/51.6–78.5	63.3 /7.7/49.9–78.4	˂.000*
Disease duration (years)	5.3/0.9/4−8	3/1.4/1−7	3.5/1.5/1−8	˂.000*
MMSE	28.21/1.5/23−30	24.03/4.3/12−30	27.5/1.9/23−30	<.000**
FDA Total score	28.0/8.84/0.00–46.0	46.36/9.26/29.0–68.0	49.60/9.01/36.0–70.0	<.000*
UPDRS III	34.3/7/ 22–48	45.17/15.1/14−64	42.6/11.6/22−58	<.012***
LEDD (mg/day)	891.3/323.5/300−1600	713.8/339.8/100−1700	758.9/314.7/400−1500	n.s.

Note

A threshold of significance was set at *p < *.01.

Abbreviations: FDA, Frenchay dysarthria assessment; LEDD, levodopa equivalent daily dose; MMSE, Mini‐Mental State Examination; MSA, multiple system atrophy; n.s., not significant; PD, Parkinson's disease; PSP, progressive supranuclear palsy; UPDRS, unified Parkinson disease rating scale.

* Significant difference between PD versus PSP, MSA

** Significant difference between PD versus PSP and MSA versus PSP

*** Significant difference between PD versus PSP

## 3 METHODS

Speech assessment (presence, type) was based on perceptual classifications of speech dimensions adapted from Darley et al. (1969a, 1969b). Frenchay Dysarthria Assessment (FDA) was used to assess speech abnormalities. The test is composed of eight categories in order: reflexes, respiration, lips, jaw, palate, larynx, tongue, and intelligibility subdivided into 28 specific vocal activities. Each of them consists of a 5‐point scale, where '0' means no speech disorder, and the higher the score, the greater the speech disorder. Next, the Mayo Clinic dysarthria classification was used (Duffy, 2005). The level of voice and speech disability was also measured using the patient self‐assessment questionnaire, the Voice Handicap Index (VHI) (Jacobson et al., 1997). A patient assesses voice impairment in three domains: functional, physical, and emotional. Each domain consists of 10 items (30 items in total), assessed on a scale of 0–4. The total VHI score reflects the severity of voice impairment as perceived by the patient and falls within the following ranges: a score of 0–30 points indicates mild dysphonia, of 31–60 points—moderate dysphonia, above 61 points—severe dysphonia (Jacobson et al., 1997; Pruszewicz, Obrębowski, Wiskirska‐Woźnica, & Wojnowski, 2004). The total VHI scores (VHI TOTAL) were taken into consideration to differentiate the severity of voice impairment in the study groups. In APS patients mixed dysarthria with hypokinetic, spastic, and ataxic components were diagnosed, and a range of severity was from moderate to severe. In most PD patients, hypokinetic dysarthria was observed. Severity has ranged from mild to moderate.

The acoustic voice analysis was performed in all subjects using the dedicated DiagnoScope Specialist software (DiagNova Technologies). Each task was done fully automatically by DiagnoScope Specialist software. Voice samples were recorded in a soundproof laboratory, with an average noise level of maximum 30 dB, using a large membrane multidirectional microphone, with bandwidth of 40 Hz–18 kHz, sensitivity 10 mV/pa, threshold sound pressure level 142 dB, and dynamic range 119 dB. During the recording, the microphone was on a support stand, at the level of the patient's mouth, at a distance of 20 cm (±5 cm). The acoustic signal was digitally processed using a 24‐bit preamplifier M‐Audio M‐Track and saved on the hard drive of a computer running on a 64‐bit operating system. The acoustic assessment has covered the voice performance module that allows evaluating voice performance and the phrase analysis module that allows evaluating words intonation. Participants have been recorded during a single session. All subjects were instructed to perform two vocal tasks, sustained phonation of the vowel/a/ as long as possible per one breath repeated three times. Only the best acoustic performance of the sample was considered. Second task phrase analysis (reading), participants were asked to read sentences at their usual rate and loudness. Phrase analysis included nine sentences that have differed intonationally. It has contained three sentences in the questioning, indicative, and imperative mood. The reading task was the same for all subjects, and the reading order was also the same. Acoustic parameters were selected and assigned to specific types of dysarthria based on the previous description of acoustic vocal assessment (Rusz et al., 2011). We evaluated dimensions observed in PD, PSP, and MSA patients including airflow insufficiency, harsh voice, mono‐pitch and mono‐loudness, strained‐strangled voice, effortful and unstable phonation with breaks and spastic aphonia, uncontrolled changes in voice pitch and volume, vocal tremor (Darley et al., 1969a, 1969b; Kluin et al., 2001; Rusz et al., 2015, 2019; Tykalova et al., 2017). Assessed parameters are listed in Table 2.

**TABLE 2:** Selected voice acoustic parameters and corresponding speech characteristics in hypokinetic, spastic, and ataxic dysarthria (Huh et al., 2015; Rusz et al., 2011, 2015, 2019; Skodda, Grönheit, Mancinelli, & Schlegel, 2013)

Deviant speech dimensions	Parameter (abbr)/unit, definition	Vocal task	Description
Hypokinetic			
Mono‐pitch	Pitch variability (F₀dev)/semitone, standard deviation of fundamental frequency determined after all basic periods	Reading	Voice inflection ability/monotone voice, emotional intonation
Reduced loudness	Acoustic energy (E)/dB, base period energy averaged over the length of the entire sample	Reading	Voice intensity, breathiness, asthenic voice
Airflow insufficiency	Maximum phonation time (MPT)/s, duration of sustained vowel phonation. Phonatory efficiency (PerfCoef)/‐, numeric parameter dependent upon voice quality and phonation length; the “better” voice and longer phonation, the higher (better) parameter value	Sustained phonation	Phonation length, respiratory support for speech and length of exhalation phase
Harsh voice	Jitter (Jitt)/%, micro perturbations of frequency. Shimmer (Shimm)/%, micro perturbations of amplitude Nonharmonic to harmonic ratio, NHR, comparison of harmonic and inharmonic sound components, amount of noise in the speech signal	Sustained phonation	Hoarseness of voice, “coated” voice
Ataxic			
Vocal tremor	Depth of fundamental frequency modulation (F₀ModDepth), the frequency of the largest spectrum component in the range of 1–20 Hz determined jointly for intervals containing phonation	Sustained phonation	Tremulous phonation
Excess pitch fluctuations	Changes in the voice pitch in the prolonged phonation period (F₀dev)/semitone	Sustained phonation	Uncontrolled changes in voice pitch
Excess loudness variations	Standard deviation of amplitude defining alterations of loudness in the prolonged phonation period (Shimm dev)/st	Sustained phonation	Uncontrolled alterations of loudness
Spastic			
Strained‐strangled voice quality	Subharmonic to harmonic ratio (S2H)/%, comparison of subharmonic and harmonic in the speech signal, nonsymmetrical motion of the vocal folds	Sustained phonation	Effortful, squeezing phonation, with a hard voice attitude
Voice breaks	Breaks (BreaksCoef)/%, continuous intervals below the phonation threshold within the intervals denoted as phonation	Sustained phonation	Phonation instability, phonation breaks
Voiceless	No phonation (NoPhonCoef)/%, ratio of total length of basic time intervals denoted as phonation and having the value of voiced parameter below minimum to the maximum phonation time	Sustained phonation	Phonation instability, spastic aphonia

### 3.1 Statistical analysis

Statistical analysis was performed using the STATISTICA 13.1 package. Normality of distribution was assessed with the Shapiro–Wilk test. The normal‐like distribution was analyzed using the one‐way ANOVA. The distribution data deviating from normality were analyzed using the Kruskal–Wallis test. Post hoc analyses were conducted using the Tukey mean difference test for the one‐way ANOVA and multiple comparisons of mean ranks for all samples tested by the nonparametric Kruskal–Wallis. Bonferroni's adjustment for multiple comparisons was used, and a significance level was set at *p* < .0125. Differences in the distribution of the qualitative data were analyzed using the Chi‐square test.

### 3.2 Ethical compliance statement

Approval for this work was obtained from the Ethics Committee of the Medical University of Warsaw, KB/6/2016. Each participant provided written, informed consent.

## 4 RESULTS

Demographic and clinical data of the patients are shown in Table 1. PD subjects were significantly younger than PSP (*p* = .00001) and MSA (*p* = .027) subjects and CG (*p* = .019), but their disease duration was significantly longer compared with PSP (*p* = .0000) and MSA subjects (*p* = .0000; Table 1). Global cognition was significantly decreased in PSP patients compared to PD (*p* = .0000) and MSA groups (*p* = .0013). In the perceptual assessment, speech disorders were found in all patients, but in PSP and MSA patients were significantly increased compared to PD (*p* = .0000). The motor scores were significantly worse in patients with PSP compared to PD (*p* = .0122), and this difference was not observed in patients with MSA.

The results of the assessed acoustic parameters in the groups are shown in Table 3. Of the speech dimensions classified as hypokinetic features, only MPT differentiated MSA and PD (*p* = .0102). The only NHR parameter did not differentiate between MSA and PD with the CG. Also, Shimmer and Perf Coef did not differentiate between PD and CG. The other parameters were significantly lower compared to the control group.

**TABLE 3:** Selected parameters of acoustic analysis in study groups and intergroup differences

Parameter	PSP *n* = 30 Mean/*SD*/range	MSA *n* = 30 Mean/*SD*/range	PD *n* = 30 Mean/*SD*/range	CG *n* = 26 Mean/*SD*/range	*p* value					
					PSP versus MSA	PSP versus PD	MSA versus PD	PSP versus CG	MSA versus CG	PD versus CG
Hypokinetic deviant speech dimensions Mono‐pitch										
F₀ dev (semitone)	0.9/0.1/0.7–1.2	1.0/0.2/0.3–1.5	1.0/0.2/0.6–1.8	1.3/0.1/1.2–1.7	n.s.	n.s.	n.s.	.0000	.0001	.0000
Reduced loudness										
E (dB)	34.8/6.1/ 25.5–50.2	33.1/4.3/19.3–39.7	33.7/5.9/24.2–45.4	41.8/6.5/32.1–55.4	n.s.	n.s.	n.s.	.007	.0000	.0004
Airflow insufficiency										
MPT (s)	12.2/5.4/4.5–23.3	10.6/5.4/2.2–26.2	16.2/6.7/3.6–32.8	22.9/4.4/15.6–32.1	n.s.	n.s.	.010	.0000	.0000	.005
Perf Coef	7.1/3.5/2.4–13.8	7.2/5.2/0.9–22.1	13.1/6.8/1.1–27.8	20.2/5.3/13.8–35.8	n.s.	n.s.	n.s.	.0000	.0000	n.s.
Harsh voice										
Jitter (%)	1.7/2.7/0.3–11.4	1.7/2.4/0.3–13.7	0.8/0.5/0.2–2.4	0.3/0.1/0.0–0.7	n.s.	n.s.	n.s.	.0000	.0000	.0000
Shimmer (%)	11.8/14.4/2.9–71.4	11.4/9.0/2.4–42.9	6.4/3.1/2.0–13.5	4.8/1.9/1.3–9.0	n.s.	n.s.	n.s.	.006	.0005	n.s.
NHR	3.8/2.5/0.8–9.2	3.3/1.9/0.5–8.7	2.9/1.9/0.7–8.7	2.1/0.8/1.0–4.8	n.s.	n.s.	n.s.	.031	n.s.	n.s.
Ataxic deviant speech dimensions Vocal tremor										
F₀ModDep	1.1/0.5/0.6–3.5	1.5/0.6/0.9–3.4	0.9/0.3/0.0–1.4	0.8/0.4/0.0–1.6	.006	n.s.	.002	n.s.	.0000	n.s.
Excess pitch fluctuations										
F₀ dev (semitone)	0.9/0.3/0.1–1.7	1.1/0.2/0.6–1.6	0.6/0.3/0.2–1.3	0.5/0.3/0.2–1.3	n.s.	n.s.	.0000	.003	.0000	n.s.
Excess loudness variations										
Shimm dev (st)	11.0/10.7/2.2–48.3	19.5/10.2/6.3–50.8	5.8/3.7/1.3–18.9	3.1/1.2/ 1.3–6.2	.012	n.s.	.0000	.0000	.0000	n.s.
Spastic deviant speech dimensions Strained‐strangled voice quality										
S2H	2.0/2.8/0.3–12.3	2.5/1.9/0.3–10.0	0.8/0.6/0.2–3.3	0.3/0.1/0.1–0.9	n.s.	n.s.	.001	.0008	.0000	n.s.
Voice breaks										
BreaksCoef (%)	0.0/0.0/0.00–0.019	0.0/0.0/0.0–0.01	0.0/0.0/0.0–0.01	0.00	n.s.	.0000	.0000	.0000	.0000	n.s.
Voiceless										
NoPhonCoef (%)	0.6/1.6/0.0–8.7	0.1/0.2/0.0–0.6	0.0/0.0/0.0–0.2	0.01/0.02/0.00–0.1	n.s.	.0000	.0008	.0000	.0000	n.s.
Perceptual speech assessment										
FDA	46.3/9.2/29.0–68.0	49.6/9.0/36.0–70.0	28.0/8.8/0.0–46.0	0.00	n.s.	.0000	.0000			
Voice self‐assessment										
VHI Total	54.3/28.4/1−102	58.1/19.8/12−92	21.0/16.7/0−56	0.00	n.s.	.0011	.0001	.0000	.0000	.0011

Note

F₀ dev standard deviation of fundamental frequency converted to semitone scale, E base period energy averaged over the length of the entire sample, voice intensity, MPT duration of sustained vowel phonation, Perf Coef phonatory efficiency, Jitter micro perturbations of frequency, Shimmer microperturbations of amplitude, NHR amount of noise in the speech signal, F₀ModDep depth of fundamental frequency modulation, vocal tremor, Shimm dev alterations of loudness in the prolonged phonation period, S2H subharmonic to harmonic ratio, BreaksCoef continuous intervals below the phonation threshold within the intervals denoted as phonation, NoPhonCoef ratio of total length of phonation to the maximum phonation time.

Abbreviations: FDA, Frenchay dysarthria assessment; n.s., not significant; VHI, Voice Handicap Index, voice self‐assessment questionnaire, *p* < .0125.

The depth of fundamental frequency modulation has been significantly increased in the MSA group compared to PSP (*p* = .006), PD (*p* = .002), and control group (*p* = .0000). No such differences were observed between PSP and PD, PSP and CG, and PD and CG. The most important changes related to the variability of the fundamental frequency (changes in voice pitch in the prolonged phonation period) were recorded in MSA patients, although patients in the group have had clinically diagnosed MSA‐P. Uncontrolled changes in voice pitch differentiated MSA from PD (*p* = .0000), MSA from CG (*p* = .0000), and PSP from CG (*p* = .003). The differences between MSA and PSP patients did not reach statistical significance. Uncontrollable changes in voice volume often observed in ataxic dysarthria were the most prominent in MSA patients. This feature differentiates MSA and PSP patients (*p* = .0124), and PD patients (*p* = .0000) and controls (*p* = .0000), and PSP patients from controls (*p* = .0000). Uncontrollable changes in voice volume did not differentiate between PSP and PD patients and PD and controls.

Strained‐strangled voice quality has been observed in APS patients. But the groups were not different. The differences reached the level of statistical significance between MSA and PD (*p* = .001), MSA and controls (*p* = .0000), and PSP and controls (*p* = .0008). A markedly increased number of subharmonic components has also observed between patients with PSP and the control group (*p* = .0008). While between PSP and PD and PSP and MSA the differences were at a similar level. Spastic features were recorded in APS patients, but the most significant changes in phonation stability, such as phonation breaks and no phonation, were observed in PSP patients. The phonation breaks coefficient (*p* = .0000) and no phonation coefficient (*p* = .0000) differentiate PSP patients from PD and controls (*p* = .0000). Furthermore, the results in MSA patients were markedly worse than in PD (Breaks Coef *p* = .0000, NoPhonCoef *p* = .0008) and controls (Breaks Coef *p* = .0000, NoPhonCoef *p* = .0000). Phonatory instability related to phonation breaks and no phonation did not differentiate APS patients. The results in PD patients and controls reached a similar level.

Voice handicap was assessed using the dedicated VHI questionnaire. The differences in the severity of voice handicap between groups were determined using the total scores. The highest total VHI scores (severe voice impairment) were found in MSA and PSP patients with no statistical differences between the two groups. The differences between APS and PD patients (PSP vs. PD, *p* = .0011; MSA vs. PD, *p* = .0001) and controls (PSP vs. CG, *p* = .0000; MSA vs. CG, *p* = .0000) were statistically significant.

**FIGURE 1:**
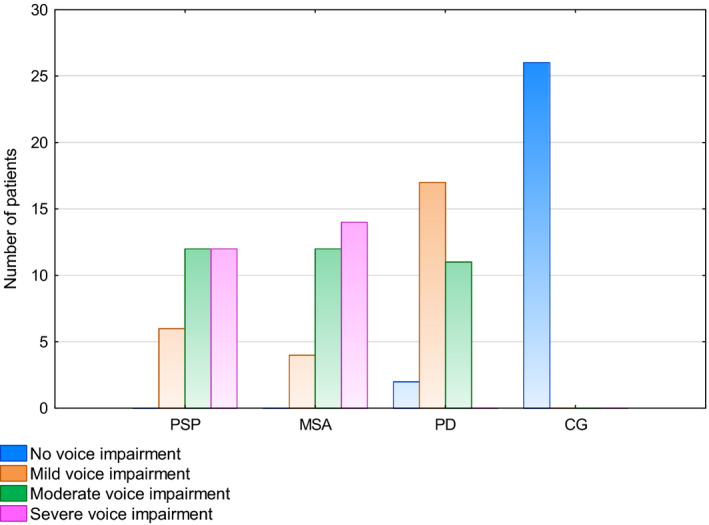
Severity of voice impairment in the study groups

The VHI TOTAL was used to assess of the severity of voice impairment in the groups (Figure 1). Out of 30 MSA patients, voice impairment was severe in 14 patients (46.67%) and moderate in 12 patients (40.0%), that is, 86.67% of the MSA group. In four MSA patients (13.33%), only voice impairment was mild. Among PSP patients, voice impairment was severe in 12 patients (40.0%), moderate in 12 (40.0%), and mild in six (20.0%). In the PD group, two patients (6.67%) had no voice impairment, 17 (56.67%) had mild voice impairment, and 11 patients (36.67%) had moderate voice impairment.

## 5 DISCUSSION

In our study, speech disorder has been observed in all patients. In the APS subjects, dysarthria has manifested as a combination of hypokinetic, spastic, and ataxic features, whereas in the PD group, mainly hypokinetic dysarthria was observed. Similar observations were found in a previous study (Darley et al., 1969a; Hartelius et al., 2006; Kluin et al., 1993, 1996, 2001; Rusz et al., 2011, 2015, 2019; Sachin et al., 2008; Skodda et al., 2011).

Hypokinetic parameters were reduced in each patient group but did not differentiate between these groups. According to Darley et al., hypokinetic dysarthria reflects extrapyramidal dysfunction (Darley et al., 1969a). Moreover, the hypokinetic components of dysarthria have strongly correlated with neuronal loss and gliosis in the substantia nigra pars compacta (Kluin et al., 2001). It is suggested that hypokinetic speech disorders are related to general motor impairment observed in PD or APS patients (Kluin et al., 2001; Rusz et al., 2019). According to the previous study, there is a relationship between the severity of speech disorder and the degree of general motor disability in patients with parkinsonian syndrome. (Huh et al., 2015; Kluin et al., 2001; Midi et al., 2008; Rusz et al., 2019; Skodda et al., 2011). Based on these results, we suppose that it may have a direct impact on the respiratory, phonatory, and articulatory system. Moreover, inappropriate propagation of acoustic wave may result at least partly in distorted phonation and articulation and hence a lower volume, and poorer clarity, and intelligibility of speech. Incomplete closure of the glottis and lower subglottal pressure affect the distribution of acoustic energy, resulting in the weakening of voice intensity. Decreased voice volume and soft speech (hypophonia) were observed in all patients and did not differentiate between PD and APS patients. However, from a clinical perspective, it is worth pointing out that acoustic energy distribution, in particular, significantly differed and thereby distinguished PD patients from controls. Hypophonia observed in this group of patients can reflect an incomplete tightening of the vocal folds and insufficient subglottal pressure, resulting in the air “escaping” through the glottis and turbulently flowing through the vocal tract, hence, decreasing the sound volume and intensity (Pinto, Chan, Guimarães, Rothe‐Neves, & Sadat, 2017; Sachin et al., 2008; Skodda et al., 2011). Voice abnormalities expose features of PD speech related to phonatory impairment and are also confirmed by some researchers (Fox & Ramig, 1997; Rusz et al., 2011). In the current study, authors show that disturbances of acoustic energy distribution in PD patients compared to controls can be attributable to bradykinesia and rigidity of intrinsic laryngeal muscles. Our theory was supported by the results of the previous study (Huh et al., 2015; Midi et al., 2008; Perez, Ramig, Smith, & Dromey, 1996; Warnecke et al., 2019). The results have shown that PD‐related dysphonia may reflect the rigidity of laryngeal muscles. Patients with PD had more severe changes in voice quality compared to controls, which were linked with larynx tremor and incomplete glottic closure. This phenomenon is common in 60% of PD patients, and the mechanism was associated with hypokinesia and rigidity of laryngeal and/or respiratory muscles (Midi et al., 2008). In other study, authors concluded that vocal fold tremor is a typical clinical feature in most of the PD patients and is caused by vertical laryngeal tremor during vocal tasks (Warnecke et al., 2019). Thus, we assume these parameters could be a useful marker for the diagnosis of PD in the early stage of the disease. Harsh voice quality was found in all patients but did not differentiate between groups of patients. It was markedly increased compared to patients and controls, except for Shimmer and NHR in PD, and NHR in MSA. Similar observations were found by Huh et al. (Huh et al., 2015). The results obtained in the present study explicitly point to the presence of mainly spastic and hypokinetic components of dysarthria and to a much lesser extent—ataxic component in PSP patients. The severity of the spastic component (phonation breaks and no phonation) was significantly increased. Spastic dysarthria associated with the damage to the corticobulbar tract results in increased spastic tension and destabilizes the function of phonatory and articulatory muscles. Speech is constrained, and forced, voice has a strained‐strangled quality and is hoarse, and phonation unstable with breaks. Speech disturbances in our PSP patients were consistent with the results of the previous study that used objective measurements (Huh et al., 2015; Rusz et al., 2015, 2019).

In MSA, speech disorders are described as mixed dysarthria with hypokinetic and ataxic components and less severe spastic features. Interestingly, although only patients with the clinical diagnosis of MSA‐P were included in the study, the ataxic component was identified in this group. It may confirm the sensitivity of the vocal apparatus to the smallest disruptions in impulsion and the role of the cerebellum and/or cortical‐pontocerebellar tract in the development of speech disorders (O'Sullivan et al., 2008; Starowicz‐Filip et al., 2017). Statistically significant differences were observed between MSA and PD patients in each ataxic and spastic parameter as well as in one hypokinetic parameter, that is, MPT. In the study reported by Huh and colleagues, the MPT parameter was affected only in female patients during the early stages of MSA and PD (Huh et al., 2015). Nonsymmetrical motion of the vocal folds (strained‐strangled voice quality), vocal tremor, significantly poorer MPT and uncontrollable changes in the voice pitch and volume allows differentiating between MSA and PD patients. The speech of MSA patients is characterized by hypophonia, dysprosody, and more nonsymmetrical mobility of the vocal folds than in PD patients, which in combination with the more pronounced changes in the voice pitch give an acoustic impression of a more severe vocal tremor. Based on a previous study (Warnecke et al., 2019), authors found that MSA patients exhibited laryngeal and pharyngeal disruptions on flexible endoscopic evaluation. All patients showed vocal fold abduction, resulting in a narrow glottic gap. Further, authors suggest that irregular arytenoid cartilage movements can be a biomarker for differentiation of MSA and PD (Warnecke et al., 2019). In another study, bilateral vocal fold motions impairment was found in 17 MSA patients, most of whom had moderate to severe bilateral vocal fold abductor restriction (Higo, Tayama, Watanabe, & Nitou, 2003). Additionally, greater phonatory instability in the form of phonation breaks and no phonation was observed in MSA patients than in PD patients. Secondary to these changes, speech clarity (intelligibility) was decreased to such an extent that their comprehensibility to those around them was dramatically reduced. In the present study, such parameters as depth of fundamental frequency modulation or uncontrollable changes in the voice volume allowed distinguishing between MSA and PSP patients. The above observation is also confirmed by Rusz et al. (Rusz et al., 2015). They conducted the acoustic analysis of 12 patients with probable PSP, 13 MSA patients, and 15 PD patients. In objective assessment, speech disorders in APS patients seemed to be a combination of hypokinetic, spastic, and ataxic components. The speech of PSP patients (83% of subjects) was described as hypokinetic and spastic (hypokinetic in 51% and spastic in 43%) The speech of MSA patients was assessed as ataxic (in 56%) and spastic (in 45%), and speech disorders were mainly in the form of ataxic dysarthria (46%) or mixed dysarthria, with variable proportions of hypokinetic, ataxic, and spastic characteristics. Speech disorders in APS patients suggest broader pathology involving the cortical structures, basal ganglia, midbrain, and cerebellum (Rusz et al., 2011, 2015). Pure hypokinetic dysarthria is observed in PD patients only. We obtained similar results in our study. In patients with PSP, mixed dysarthria was diagnosed with a predominance of hypokinetic and spastic components, in patients with MSA speech disorders were described as hypokinetic‐atactic‐spastic dysarthria, while patients with PD have hypokinetic dysarthria.

In the self‐assessment of voice handicap, voice impartment was more frequent and severe in APS patients that in PD patients. In 86.67% of MSA patients, speech disorders were reported as severe and moderate. Among PSP patients, 80% reported moderate and severe voice impairment, whereas in most PD patients (56.67%) voice impartment was mild.

## 6 CONCLUSIONS

Our study has limitations. First of all, groups are not gender‐balanced. Therefore, a gender impact on the results cannot be excluded. It is suggested that the discrepancy may be due to the anatomical structure of the larynx, and various weights of disease change on neural reflex for speech generation (Huh et al., 2015). On the other hand, an additional subanalysis of acoustic parameters in the control group was performed. The results clearly indicate that there are no differences in the assessed parameters between male and female. The acoustic assessment was focused only on phonation and reading tasks. Further speech dimensions, for example, speech rate and rhythm, diadochokinesis, and articulation should be investigated.

Database search for studies on speech assessment in PD and APS patients found mainly studies focused on perceptual speech parameters. The acoustic analysis allows quantitative and objective assessment of voice parameters. It is a very sensitive, objective, and noninvasive tool, providing quantitative data essential for objective measurement of speech disorders; hence, it is a more accurate tool than perceptual assessment and may be useful in the differential diagnosis of parkinsonian syndromes. Moreover acoustic analysis can provide feedback of disease progression and treatment response. Thus, further research is needed using more accurate measurement scales to assess voice parameters in specific speech disorders to learn more about the neuropathology and mechanisms of their development in PD and APS.

## CONFLICT OF INTEREST

The authors declare no competing financial interests.

## AUTHORS CONTRIBUTION

Renata Kowalska‐Taczanowska, MSc involved in substantial contributions to conception and design, acquisition, analysis and interpretation of data, and drafting the manuscript. Andrzej Friedman, MD, PhD, Chair and Professor of Neurology involved in substantial contributions to conception and design, acquisition, analysis and interpretation of data, revising the manuscript critically for important intellectual content, and approval of the version to be published. Dariusz Koziorowski, MD, PhD involved in substantial contributions to conception and design, acquisition, analysis, and interpretation of data, revising the manuscript critically for important intellectual content, and approval of the version to be published.

## Data Availability

The data supporting the results in this study are available from the corresponding author upon reasonable request.

## References

[brb31700-bib-0001] Berardelli, A. , Wenning, G. K. , Antonini, A. , Berg, D. , Bloem, B. R. , Bonifati, V. , … Vidailhet, M. (2013). EFNS/MDS‐ES recommendations for the diagnosis of Parkinson's disease. European Journal of Neurology, 20, 16–34. 10.1111/ene.12022 23279440

[brb31700-bib-0002] Darley, F. L. , Aronson, A. E. , & Brown, J. R. (1969a). Differential diagnostic patterns of dysarthria. Journal of Speech and Hearing Research, 12(2), 246–269. 10.1044/jshr.1202.246 5808852

[brb31700-bib-0003] Darley, F. L. , Aronson, A. E. , & Brown, J. R. (1969b). Clusters of deviant speech dimensions in the dysarthrias. Journal of Speech and Hearing Research, 12(3), 462–469. 10.1044/jshr.1203.462 5811846

[brb31700-bib-0005] Duffy, J. R. (2005). Motor speech disorders: Substrates, differential diagnosis and management (2nd ed.). New York, NY: Mosby.

[brb31700-bib-0006] Fox, C. , & Ramig, L. O. (1997). Vocal sound pressure level and self‐perception of speech and voice in men and women with idiopathic Parkinson disease. American Journal of Speech‐Language Pathology, 6(2), 85–94. 10.1044/1058-0360.0602.85

[brb31700-bib-0007] Gilman, S. , Wenning, G. K. , Low, P. A. , Brooks, D. J. , Mathias, C. J. , Trojanowski, J. Q. , … Vidailhet, M. (2008). Second consensus statement on the diagnosis of multiple system atrophy. Neurology, 71(9), 670–676. 10.1212/01.wnl.0000324625.00404.15 18725592 PMC2676993

[brb31700-bib-0008] Hartelius, L. , Gustavsson, H. , Astrand, M. , & Holmberg, B. (2006). Perceptual analysis of speech in multiple system atrophy and progressive supranuclear palsy. Journal of Medical Speech‐Language Pathology, 14(4), 241–247.

[brb31700-bib-0009] Higo, R. , Tayama, N. , Watanabe, T. , & Nitou, T. (2003). Vocal fold motion impairment in patients with multiple system atrophy: Evaluation of its relationship with swallowing function. Journal of Neurology, Neurosurgery and Psychiatry, 74(7), 982–984. 10.1136/jnnp.74.7.982 PMC1738548 12810801

[brb31700-bib-0010] Hlavnička, J. , Tykalová, T. , Čmejla, R. , Klempíř, J. , Růžička, E. , & Rusz, J. (2017). Dysprosody differentiate between Parkinson's disease, progressive supranuclear palsy, and multiple system atrophy. Conference paper: Interspeech 2017, Stockholm, Sweden.

[brb31700-bib-0011] Ho, A. K. , Iansek, R. , Marigliani, C. , Bradshaw, J. , & Gates, S. (1999). Speech impairment in large sample of patients with Parkinson's disease. Behavioural Neurology, 11, 131–137. 10.1155/1999/327643 22387592

[brb31700-bib-0012] Höglinger, G. U. , Respondek, G. , Stamelou, M. , Kurz, C. , Josephs, K. A. , Lang, A. E. , … Litvan, I. (2017). Clinical diagnosis of progressive supranuclear palsy: The Movement Disorder Society criteria. Movement Disorders, 32(6), 853–864. 10.1002/mds.26987 28467028 PMC5516529

[brb31700-bib-0013] Huh, Y. E. , Park, J. , Suh, M. K. , Lee, S. E. , Kim, J. , Jeong, Y. , … Cho, J. W. (2015). Differences in early speech patterns between Parkinson variant of multiple system atrophy and Parkinson's disease. Brain and Language, 147, 14–20. 10.1016/j.bandl.2015.04.007 25997172

[brb31700-bib-0014] Jacobson, B. H. , Johnson, A. , Grywalski, C. , Silbergleit, A. , Jacobson, G. , Benninger, M. S. , & Newman, C. W. (1997). The Voice Handicap Index (VHI): Development and Validation. American Journal of Speech‐Language Pathology, 6(3), 66–70. 10.1044/1058-0360.0603.66

[brb31700-bib-0015] Kim, J.‐H. , & McCann, C. M. (2015). Communication impairments in people with progressive supranuclear palsy: A tutorial. Journal of Communication Disorders, 56, 76–87. 10.1016/j.jcomdis.2015.06.002 26184056

[brb31700-bib-0016] Kluin, K. J. , Foster, N. L. , Berent, S. , & Gilman, S. (1993). Perceptual analysis of speech disorders in progressive supranuclear palsy. Neurology, 43(3), 563–566. 10.1212/WNL.43.3_Part_1.563 8451002

[brb31700-bib-0017] Kluin, K. J. , Gilman, S. , Foster, N. L. , Sima, A. A. F. , D'Amato, C. J. , Bruch, L. A. , … Johanns, J. (2001). Neuropathological correlates of dysarthria in progressive supranuclear palsy. Archives of Neurology, 58(2), 265–269. 10.1001/archneur.58.2.265 11176965

[brb31700-bib-0018] Kluin, K. J. , Gilman, S. , Lohman, M. , & Junck, L. (1996). Characteristics of the dysarthria of multiple system atrophy. Archives of Neurology, 53(6), 545–548. 10.1001/archneur.1996.00550060089021 8660157

[brb31700-bib-0019] Logemann, J. A. , Fisher, H. B. , Boshes, B. , & Blonsky, E. R. (1978). Frequency and cooccurrence of vocal tract dysfunction in the speech of a large sample of Parkinson patients. Journal of Speech and Hearing Disorder, 43, 47–57. 10.1044/jshd.4301.47 633872

[brb31700-bib-0020] Midi, I. , Dogan, M. , Koseoglu, M. , Can, G. , Sehitoglu, M. A. , & Gunal, D. I. (2008). Voice abnormalities and their relation with motor dysfunction in Parkinson's disease. Acta Neurologica Scandinavica., 117, 26–34. 10.1111/j.1600-0404.2007.00965.x 18031561

[brb31700-bib-0021] Nath, U. , Ben‐Shlomo, Y. , Thomson, R. G. , Lees, A. J. , & Burn, D. J. (2003). Clinical features and natural history of progressive supranuclear palsy – A clinical cohort study. Neurology, 60(6), 910–916. 12654952 10.1212/01.wnl.0000052991.70149.68

[brb31700-bib-0022] Osaki, Y. , Ben‐Shlomo, Y. , Lees, A. J. , Daniel, S. E. , Colosimo, C. , Wenning, G. K. , & Quinn, N. (2004). Accuracy of clinical diagnosis of progressive supranuclear palsy. Movement Disorders, 19(2), 181–189. 10.1002/mds.10680 14978673

[brb31700-bib-0023] O'Sullivan, S. S. , Massey, L. A. , Williams, D. R. , Silveira‐Moriyama, L. , Kempster, P. A. , Holton, J. L. , … Lees, A. J. (2008). Clinical outcomes of progressive supranuclear palsy and multiple system atrophy. Brain, 131(5), 1362–1372. 10.1093/brain/awn065 18385183

[brb31700-bib-0024] Perez, K. S. , Ramig, L. O. , Smith, M. E. , & Dromey, C. (1996). The Parkinson larynx: Tremor and videostroboscopic findings. Journal of Voice, 10(4), 354–361. 10.1016/S0892-1997(96)80027-0 8943139

[brb31700-bib-0025] Pinto, S. , Chan, A. , Guimarães, I. , Rothe‐Neves, R. , & Sadat, J. (2017). A cross‐linguistic perspective to the study of dysarthria in Parkinson's disease. Journal of Phonetics, 64, 156–167. 10.1016/j.wocn.2017.01.009

[brb31700-bib-0026] Poewe, W. (2008). Non‐motor symptoms in Parkinson's disease. European Journal of Neurology, 15(1), 14–20. 10.1111/j.1468-1331.2008.02056.x 18353132

[brb31700-bib-0027] Pruszewicz, A. , Obrębowski, A. , Wiskirska‐Woźnica, B. , & Wojnowski, W. (2004). Complex voice assessment‐Polish version of the Voice Handicap Index (VHI). Otolaryngologia Polska, 58(3), 547–549. PMID:15311601. 15311601

[brb31700-bib-0028] Rusz, J. , Bonnet, C. , Klempir, J. , Tykalova´, T. , Baborova´, E. , Novotny´, M. , … Růžička, E. (2015). Speech disorders reflect differing pathophysiology in Parkinson's disease, progressive supranuclear palsy and multiple system atrophy. Journal of Neurology, 262(4), 992–1001. 10.1007/s00415-015-7671-1 25683763

[brb31700-bib-0029] Rusz, J. , Cmejla, R. , Ruzickova, H. , & Ruzicka, E. (2011). Quantitative acoustic measurements for characterization of speech and voice disorders in early untreated Parkinson's disease. Journal of the Acoustical Society of America, 129(1), 350–367. 10.1121/1.3514381 21303016

[brb31700-bib-0030] Rusz, J. , Tykalowa, T. , Salerno, G. et al (2019). Distinctive speech signature in cerebellar and parkinsonian subtypes of multiple system atrophy. Journal of Neurology, 266, 1394–1404. 10.1007/s00415-019-09271-7 30859316

[brb31700-bib-0031] Sachin, S. , Schukla, G. , Goyal, V. , Singh, S. , Aggarval, V. , & Gureshkumar, B. M. (2008). Clinical speech impairment in Parkinson's disease, progressive supranuclear palsy and multiple system atrophy. Neurology India, 56(2), 122–126. 18688134 10.4103/0028-3886.41987

[brb31700-bib-0032] Schrag, A. , Ben‐Shlomo, Y. , & Quinn, N. P. (1999). Prevalence of progressive supranuclear palsy and multiple system atrophy: A cross‐sectional study. Lancet, 354, 1771–1775. 10.1016/S0140-6736(99)04137-9 10577638

[brb31700-bib-0034] Skodda, S. , Grönheit, W. , Mancinelli, N. , & Schlegel, U. (2013). Progression of voice and speech impairment in the course of Parkinson's disease: A Longitudinal Study. Parkinson's Disease, 2013, 1–8. 10.1155/2013/389195 PMC3872441 24386590

[brb31700-bib-0035] Skodda, S. , Gronheitet, W. , & Schlegel, U. (2012). Instability of syllable repetition in progressive supranuclear palsy. Journal of Neural Transmission, 119(4), 457–462. 10.1007/s00702-011-0737-z 22065210

[brb31700-bib-0036] Skodda, S. , Visser, W. , & Schlegel, U. (2011). Acoustical analysis of speech in progressive supranuclear palsy. Journal of Voice, 25(6), 725–731. 10.1016/j.jvoice.2010.01.002 20457507

[brb31700-bib-0037] Starowicz‐Filip, A. , Chrobak, A. A. , Moskała, M. , Krzyżewski, R. M. , Kwinta, B. , Kwiatkowski, S. , … Zielińska, D. (2017). The role of the cerebellum in the regulation of language functions. Psychiatria Polska, 51(4), 661–671. 10.12740/PP/68547 28987056

[brb31700-bib-0038] Tison, F. , Yekhlef, F. , Chrysostome, V. , & Sourgen, C. (2000). Prevalence of multiple system atrophy. Lancet, 355, 495–496. 10.1016/S0140-6736(00)82050-4 10841152

[brb31700-bib-0039] Tykalova, T. , Rusz, J. , Klempir, J. , Cmejla, R. , & Ruzicka, E. (2017). Distinct patterns of imprecise consonant articulation among Parkinson's disease, progressive supranuclear palsy and multiple system atrophy. Brain & Language, 165, 1–9. 10.1016/j.bandl.2016.11.005 27894006

[brb31700-bib-0040] Warnecke, T. , Vogel, A. , Ahring, S. , Gruber, D. , Heinze, H.‐J. , Dziewas, R. , … Gandor, F. (2019). The shaking palsy of the larynx‐potential biomarker for multiple system atrophy: A pilot study and literature review. Frontiers in Neurology, 10, 241 10.3389/fneur.2019.00241 30972002 PMC6443854

